# Diagnostic accuracy of smear microscopy, mycobacterial culture, and GeneXpert MTB/RIF assay for diagnosis of subclinical tuberculosis: a retrospective multicenter study

**DOI:** 10.1128/spectrum.01888-24

**Published:** 2025-03-31

**Authors:** Jin Shi, Yanhong Yu, Bo Li, Yuanyuan Shang, Cong Yao, Weicong Ren, Shanshan Li, Mengqiu Gao, Yu Pang

**Affiliations:** 1 Department of Tuberculosis, Beijing Chest Hospital, Capital Medical University/Beijing Tuberculosis and Thoracic Tumor Research Institute, Beijing, China; 2 Tuberculosis Laboratory, Shenyang Tenth People’s Hospital/Shenyang Chest Hospital, Shenyang, China; 3 Department of Tuberculosis, Beijing Center for Disease Prevention and Control, Beijing, China; 4 Department of Bacteriology and Immunology, Beijing Chest Hospital, Capital Medical University/Beijing Tuberculosis and Thoracic Tumor Research Institute, Beijing, China; Weill Cornell Medicine, New York, New York, USA

**Keywords:** tuberculosis, subclinical, asymptomatic, prevalence, diagnostic methods

## Abstract

Subclinical tuberculosis (TB) challenges public health interventions. This retrospective multicenter study aimed to evaluate the diagnostic efficacy of smear microscopy, mycobacterial culture, and GeneXpert MTB/RIF assay (Xpert) for subclinical TB and identify its proportion and risk factors in China. Among 560 participants diagnosed with TB, 309 (55.18%) patients had active TB, and 251 (44.82%) had subclinical TB. For subclinical TB diagnosis, smear microscopy, mycobacterial culture, and Xpert had detection rates of 11.5%, 40.6%, and 40.6%, respectively, which were significantly lower than those for active TB (25.2%, 59.2%, and 63.7% respectively). Conversely, interferon-γ release assays (IGRA) showed similar detection rates for both subclinical (85.7%) and active TB (84.1%). Age groups of 40–60 years and under 40 years, as well as employed individuals had higher risks of subclinical TB. To conclude, about 45% of TB patients were subclinical in our study. Microbiologic tests had lower diagnostic sensitivities for subclinical TB than for active TB, while IGRA positivity rates were comparable between asymptomatic and symptomatic TB patients, highlighting the potential of antigen-specific immune response in the diagnosis of these asymptomatic individuals. Further clinical trials are warranted to investigate the diagnostic flow chart in individuals with subclinical TB.

**IMPORTANCE:**

Subclinical tuberculosis (TB) poses a major challenge to public health interventions, especially in the wake of the COVID-19 pandemic. However, a substantial proportion of individuals have absence or non-recognized symptoms, which put great pressure on clinical diagnosis. In this study, we conducted a retrospective multicenter study to assess the performance of smear microscopy, mycobacterial culture, and GeneXpert MTB/RIF (Xpert) in diagnosis of subclinical TB. We also aimed to determine the proportion and risk factors of subclinical TB in China in order to accelerate progress towards global milestones and targets for the End TB Strategy.

## INTRODUCTION

Tuberculosis (TB), caused by *Mycobacterium tuberculosis* (MTB) complex, remains a major public health concern worldwide (1). Globally, an estimated 10.6 million people develop active TB, and nearly 1.3 million people die from the disease (2). Although there had been a slight decline in TB incidence over the previous years, there is a large gap between the unsatisfactory TB control programme performance and End TB Strategy goals. Indeed, the COVID-19 pandemic has emphasized the fragility of TB services and has led to a reversal in the efforts made in combating TB (3, 4). In order to accelerate progress towards global milestones and the target for the End TB Strategy, multisectoral collaboration is needed to reduce the prevalence of major risk factors for infection and disease (5).

An advanced public health response against TB requires a better understanding of the microbiological and clinical spectrum of TB infection (6). Unfortunately, TB infection is complicated by diverse clinical symptoms or radiological abnormalities (7). However, in addition to the presence of marked clinical symptoms, such as cough, fever, or night sweats, a substantial proportion of individuals have a disease caused by MTB with the absence or unrecognized symptoms, termed subclinical TB (8). Existing data increasingly show that subclinical TB contributes a large fraction of disease burden at the population level (9). Moreover, TB transmission occurs within the community during the subclinical period, emphasizing that finding these individuals is of great importance to achieve major reductions in TB incidence by 2035 (8).

Early confirmation of the diagnosis of subclinical TB is the first step to identify infection sources and thereby interrupt transmission (10). Due to the minimal bacterial emission of these asymptomatic individuals, it is a challenging problem. More importantly, the majority of previous studies estimated the diagnostic performance of multiple tools using predictive modeling, but the accuracy of routine diagnostic assays was poorly known in clinical practice (11–13). In this study, we conducted a retrospective multicenter study to assess the performance of smear microscopy, mycobacterial culture, and GeneXpert MTB/RIF assay (Xpert) in the diagnosis of subclinical TB. We also aimed to determine the proportion and risk factors of subclinical TB in China.

## MATERIALS AND METHODS

### Study design

From November to December 2023, a total of 560 patients reported in the electronic recording and reporting system for tuberculosis at three TB specialized hospitals, namely Beijing Chest Hospital, Shenyang Chest Hospital, and Beijing Research Institute for Tuberculosis Control, were retrospectively enrolled in our study. Patients with abnormal radiological features and/or clinical symptoms indicating pulmonary TB were referred to these hospitals to make a final diagnosis of TB. Routinely, sputum, and peripheral blood samples were collected for laboratory testing. For the microbiological assays, all subclinical tuberculosis patients provided bronchoalveolar lavage fluid (BALF) specimens, while active tuberculosis patients exclusively submitted sputum samples, and none of them provided BALF samples. The laboratory results of patients diagnosed with active TB were further reviewed to exclude patients without laboratory results on respiratory specimens. Finally, a total of 560 tuberculosis patients were enrolled from the three hospitals, including 227 cases from Beijing Chest Hospital, 245 cases from Shenyang Chest Hospital, and 88 cases from Beijing Research Institute for Tuberculosis Control. Clinical data were obtained from hospital electronic medical records, including demographic characteristics, comorbidities, and clinical symptoms. This study was approved by the Ethics Committee of Beijing Chest Hospital, Capital Medical University. Given that all data were kept anonymous during analysis, a waiver for informed consent was obtained.

### Definitions

Active TB disease was defined as persons presenting clinical symptoms, along with radiological abnormalities and/or microbiologic evidence of active TB disease, including definitive and clinically diagnosed TB. The definitive TB cases were defined as those with positive culture and/or Xpert results, while the clinically diagnosed TB was defined as those without positive laboratory results suggestive of the presence of tubercle bacilli but with clinical improvement after anti-TB treatment. Subclinical TB disease was defined as individuals without any TB-associated clinical symptoms, such as cough, fever, night sweats, hemoptysis, weight loss, and chest pain, but presenting abnormalities that could be detected using radiological and/or microbiological assays (14). Its definition is similar to that of latent tuberculosis. However, latent tuberculosis is characterized by the lack of radiological and microbiological evidence of active tuberculosis disease. In contrast, subclinical tuberculosis patients show certain degrees of radiological changes, which may include small nodules, mild infiltrates, or other early-stage alterations in the lung parenchyma. Microbiologically, subclinical tuberculosis patients may have a positive result from at least one of these microbiological assays. Moreover, patients will not be finally given a diagnosis of subclinical tuberculosis until positive changes are shown in the results of radiological and/or microbiological assays after 3 months of anti-tuberculosis treatment.

### Microscopy and culture of the respiratory specimens

Fluorescent smear microscopy was conducted on the respiratory specimens as previously described (15). Additionally, 2 mL of respiratory specimens was decontaminated with N-acetyl-L-cysteine-NaOH-Na citrate at a final concentration of 1.5%. Then, the respiratory specimens were incubated for 15 min at room temperature and neutralized with phosphate-buffered saline (PBS) (pH = 7.4). After centrifugation at 4,000×*g* for 15 min, the sediments were resuspended in 2 mL PBS buffer. Then, 500 µL of the resuspension was inoculated into MGIT (Mycobacterium Growth Indicator Tube) liquid medium and incubated in a BACTEC MGIT 960 mycobacterial detection instrument (Becton Dickinson, NJ, USA). The growth of mycobacteria was monitored by the instrument, and samples without growth at 42 days were classified as negative.

### GeneXpert MTB/RIF assay

Xpert was used on the respiratory specimens in accordance with the manufacturer’s instruction (16). Briefly, 1 mL of each respiratory specimen was mixed with 2 mL of Xpert sample reagent (mixture of NaOH and isopropanol) in the Falcon tube. After incubation at room temperature for 15 min, 2 mL of digested sample was transferred to the labeled cartridge and loaded onto the Xpert machine. Results reported by the Xpert system were presence or absence of tubercle bacilli, with semi-quantified bacillary load as high, medium, low, or very low.

### IFN-γ release assays

Six milliliters of venous blood samples was collected from each subject. After the blood samples were delivered to the laboratory, peripheral blood mononuclear cells (PBMCs) were isolated from them using lymphocyte separation medium (TBD, Tianjin, China). The isolated PBMCs at a density of 2.5 × 10⁶ cells/mL were incubated with the ESAT-6–CFP-10-Rv1985c fusion protein (as the specific antigen of *Mycobacterium tuberculosis*), phytohemagglutinin (PHA, as a positive control), and negative control culture medium at 37°C for 16–20 h. Subsequently, the cell culture supernatants were collected. Fifty microliters of the supernatant was used to analyze the concentration of interferon-γ by enzyme-linked immunosorbent assay. According to the manufacturer’s instructions (WanTai BioPharm, Beijing, China), the result was defined as the value of the tuberculosis antigen (T) minus that of the negative control (N), representing the level of cytokines stimulated by *Mycobacterium tuberculosis*-specific antigens. That is, when (T - N) ≥14 and *N* < 4, the result was judged as positive. In this way, we could determine whether the patient had a specific T-cell immune response against *Mycobacterium tuberculosis*.

### Statistical analysis

Statistical analysis was performed using SPSS version 22.0 (IBM Corp., Armonk, NY, USA). Categorical variables were presented as frequencies and percentages, while continuous variables were expressed as mean ± standard deviation (SD) or median with interquartile range (IQR) depending on their distribution. The χ test or Fisher’s exact test was used to compare categorical variables, while Student’s *t*-test was applied for normally distributed continuous variables and Mann–Whitney U test for non-normally distributed ones. Logistic regression analysis was conducted to identify predictors of TB disease, with adjustment for potential confounders. The odds ratios (ORs) and 95% confidence intervals (CIs) were calculated to assess the strength of association. A *P*-value < 0.05 was considered statistically significant.

## RESULTS

### Baseline characteristics and comorbidities in active and subclinical tuberculosis cases

In this cross-sectional study involving 560 participants, we analyzed the baseline characteristics of 309 (55.2%) patients with active tuberculosis (TB) and 251 (44.8%) with subclinical TB (Table 1). The median age of active TB patients was 55 years (IQR, 37–68), significantly different from that of the subclinical TB group, which was 44 years (IQR, 30–62) (*P* < 0.001). In terms of occupational status, the active TB group had a higher proportion (77.7%) of unemployed individuals, whereas the subclinical TB group had a higher proportion (31.9%) of employed individuals, and this difference being statistically significant (*P* < 0.001).

**TABLE 1:** Baseline characteristics of enrolled active and subclinical TB cases

Variables	All patients (*n* = 560)	Active TB disease (*n* = 309, 55%)	Subclinical TB disease (*n* = 251, 45%)	*P*-values^*a*^
Age	51.50 [33.00, 66.00]^*b*^	55.00 [37.00, 68.00]^*b*^	44.00 [30.00, 62.00]^*b*^	<0.001
Gender				0.664
Male	348 (62.14%)	195 (63.11%)	153 (60.96%)	
Female	212 (37.86%)	114 (36.89%)	98 (39.04%)	
Residents				
Urban	427 (76.25%)	235 (76.05%)	192 (76.49%)	0.982
Rural	133 (23.75%)	74 (23.95%)	59 (23.51%)	
Career				<0.001
Unemployment	411 (73.39%)	240 (77.67%)	171 (68.13%)	
Employee	149 (26.61%)	69 (22.33%)	80 (31.87%)	
Comorbidity				>0.05
Diabetes mellitus	98 (17.50%)	53 (17.15%)	45 (17.93%)	
Cancer	19 (3.39%)	9 (2.91%)	10 (3.98%)	
COPD	13 (2.32%)	7 (2.27%)	6 (2.39%)	
Bronchiectasis	12 (2.14%)	8 (2.59%)	4 (1.59%)	
CKD	12 (2.14%)	7 (2.27%)	5 (1.99%)	
Autoimmune Disease	11 (1.96%)	9 (2.91%)	2 (0.80%)	
Hepatitis	8 (1.43%)	6 (1.94%)	2 (0.80%)	
Asthma	2 (0.36%)	2 (0.65%)	0 (0.00%)	

^*a*^ Wilcoxon rank sum test; Pearson’s Chi-squared test; Fisher’s exact test.

^*b*^ Median [IQR]; n (%).

There was no significant gender difference between the two groups, with males accounting for 63.1% in the active TB group and 61.0% in the subclinical TB group, and females making up 36.9% and 39.0%, respectively. The distribution of residential status also showed no significant difference, with urban residents making up 76.2% of the total (76.1% and 76.5% in each group). Regarding comorbidities, the prevalence of autoimmune diseases was slightly higher in patients with active TB (2.9%) than in patients with subclinical TB (0.8%), but this difference did not reach statistical significance (*P* > 0.05). These findings suggest that age and occupational status are important factors associated with TB activity, while the impact of comorbid autoimmune diseases requires further validation with larger sample sizes. Residence status, gender, and other comorbidities did not appear to influence disease status.

### Detection rates of diagnostic methods for tuberculosis

**Fig 1:**
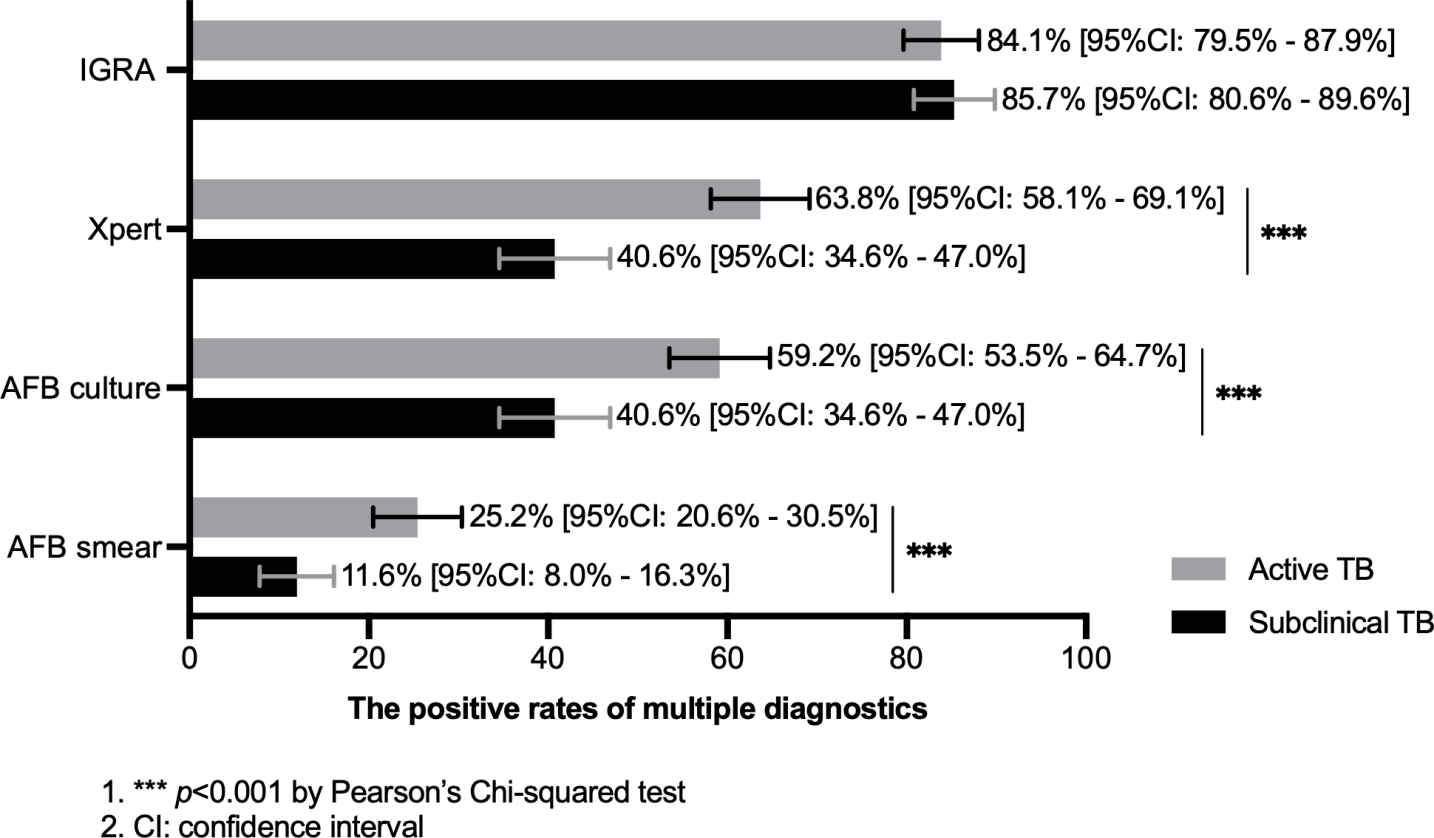
The positive rates of multiple diagnostics for detecting subclinical and active TB. The positive rates of multiple diagnostics (bars: left side *y*-axis) in subclinical TB cases (black bars) and active TB cases (gray bars). *** *P* < 0.001 by Pearson’s Chi-squared test. AFB = acid fast bacillus; IGRA = interferon-gamma release assay, CI = confidence interval.

In the comparative analysis of diagnostic methods for subclinical and active tuberculosis (TB), significant differences in detection rates were observed (Fig. 1). The acid-fast bacillus (AFB) smear showed a detection rate of 11.6% (8.0%–16.3%) for subclinical TB, substantially lower than that of 25.2% (95% CI: 20.6%–30.5%) for active TB, indicating a reduced sensitivity for subclinical cases. AFB culture detected 40.64% (95% CI: 34.6%–47.0%) of subclinical TB cases and 59.2% (95% CI: 53.5%–64.7%) for active TB, thus highlighting a higher diagnostic yield for active TB. The Xpert had a detection rate of 40.6% (95% CI: 34.6%–47.0%) for subclinical TB and 63.8% (95% CI: 58.1%–69.1%) for active TB, showing a significant difference in performance. However, interferon-γ release assays (IGRA) yielded comparable detection rates for both subclinical (85.7% [95% CI: 80.6%–89.6%]) and active TB (84.1% [95% CI: 79.5%–87.9%]).

The bacterial load detected by Xpert also showed significant differences between the two groups of patients (Table 2). Among the 560 participants, 46.6% had negative test results. Notably, the negative rate was higher in subclinical TB patients (59.4%) compared with active TB patients (36.3%). Regarding the positive cases, extremely low and low bacterial loads were more common in subclinical TB patients. In the subclinical TB group, 46.1% of the positive cases had extremely low bacterial loads, while in the active TB group, the proportion was 26.4%. For low bacterial loads, the proportion in the subclinical TB group was 32.3%, lower than 35.0% in the active TB group. However, medium and high bacterial loads were more prevalent in active TB patients. In terms of medium bacterial loads, 31.0% of the active TB patients had such levels compared with 16.7% of the subclinical TB patients. High bacterial loads were present in 7.6% of the active TB group compared with 4.9% of the subclinical group. These data clearly demonstrate the distinct bacterial load characteristics between active and subclinical TB patients.

**TABLE 2:** Different Xpert bacterial loads in patients enrolled with active or subclinical TB

Variables	All patients (*n* = 560)^*a*^	Active TB disease (*n* = 309, 55%)^*a*^	Subclinical TB disease (*n* = 251, 45%)^*a*^	*P*-values^*b*^
Xpert test result				<0.001
Negative	261 (46.61%)	112 (36.25%)	149 (59.36%)	
Extremely low	99 (17.68%)	52 (16.83%)	47 (18.73%)	
Low	102 (18.21%)	69 (22.33%)	33 (13.15%)	
Mediumm	78 (13.93%)	61 (19.74%)	17 (6.77%)	
High	20 (3.57%)	15 (4.85%)	5 (1.99%)	

^*a*^ n (%).

^*b*^ Pearson’s Chi-squared test.

**Fig 2:**
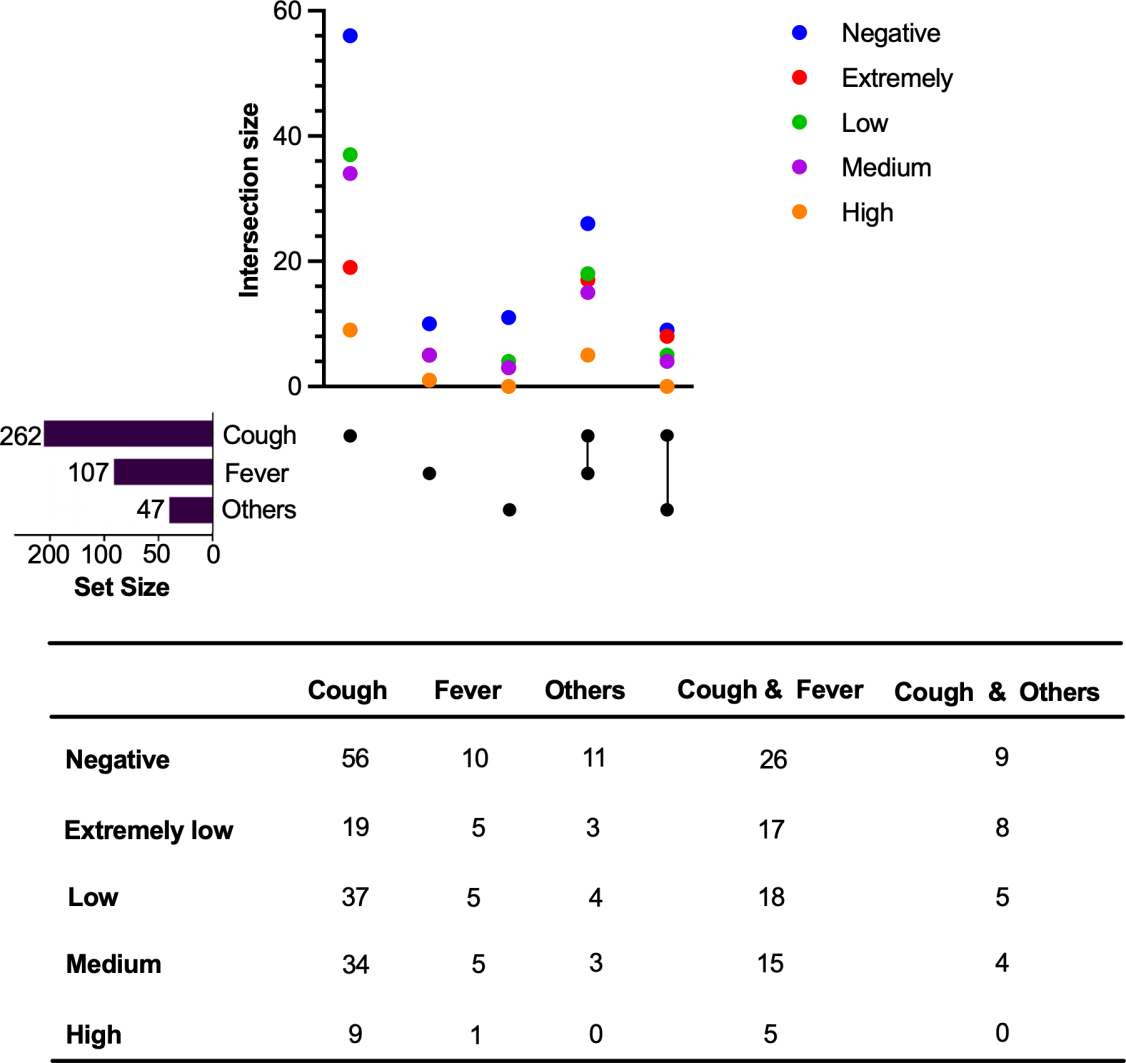
The intersection size of symptoms and Xpert’s bacterial load in active TB. This figure presents a comprehensive overview of the relationship between symptoms and the bacterial load detected by Xpert in active tuberculosis (ATB) cases. The *y*-axis represents the number of cases of patients with different symptoms detected at different Xpert bacterial load levels, namely “Negative,” “Extremely low,” “Low,” “Medium,” and “High.” The *x*-axis lists a range of symptoms and symptom combinations, including “Only Cough,” “Only Fever,” “Others” (other TB-related symptoms), “Cough combined with Fever,” and “Cough combined with Others.” Each cell in the grid represents the number of ATB cases that exhibit a particular set of symptoms at a given bacterial load level. Different levels of bacterial load are marked with different colored dots: blue represents “Negative” bacterial load, green represents “Extremely low” bacterial load, yellow represents “Low” bacterial load, orange represents “Medium” bacterial load, and purple represents “High” bacterial load. For detailed data, see the table below Fig. 2.

Additionally, we analyzed the association between symptoms and Xpert’s bacterial load (Fig. 2). “Cough” and “fever” are the most common accompanying symptoms of TB, and they are associated with different bacterial loads detected by Xpert. However, some active TB cases with cough and fever are still negative for bacterial load. Notably, the “other” category, which included chest pain, hemoptysis, weight loss, and other atypical findings, had similarly weak associations with the bacterial loads detected by Xpert. These findings highlight the urgent need to develop more sensitive and specific diagnostic methods.

### Factors associated with subclinical TB

**Fig 3:**
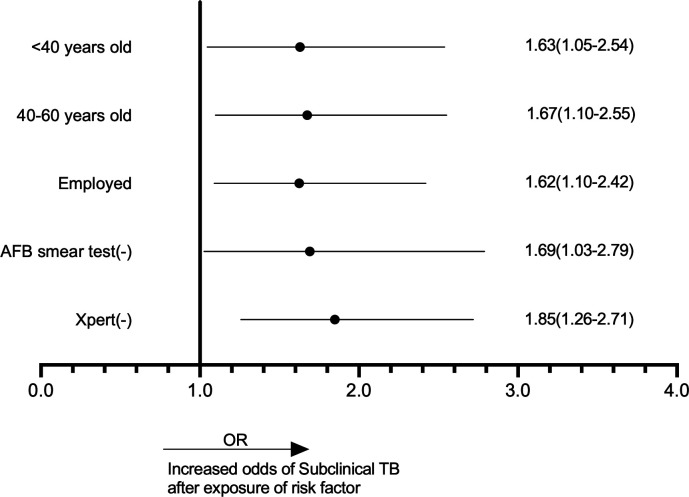
Multivariate regression analysis for factors associated with subclinical TB compared with active TB. The dots denote the summary effect sizes from random effects models, and the lines represent 95% confidence intervals for all studies. OR = odds ratio.

First, we carried out a univariate analysis to identify the potential factors related to subclinical tuberculosis and to eliminate the influence of confounding factors on the correlation (Fig. S1). Then, the factors with significant correlation in the univariate analysis were further subjected to multivariate regression analysis to reveal significant factors differentiating subclinical TB from active TB (Fig. 3). Age proved to be a pivotal factor, with individuals aged 40–60 years and those under 40 years showing increased odds of subclinical TB, with odds ratios (OR) of 1.67 (95% CI: 1.10–2.55) and 1.63 (95% CI: 1.05–2.54), respectively, compared with the reference group aged over 60 years. Employment status was also influential; employed persons had higher odds of subclinical TB, with an OR of 1.62 (95% CI: 1.10–2.42). Diagnostic test outcomes further correlated with subclinical TB; AFB smear and Xpert results were associated with increased odds of subclinical TB, with ORs of 1.69 (95% CI: 1.03–2.79) and 1.85 (95% CI: 1.26–2.71), respectively. All factors were statistically significant, with *P*-values < 0.05.

## DISCUSSION

Subclinical pulmonary tuberculosis represents a key obstacle to the elimination of TB. Our results showed that approximately 45% of TB patients were subclinical by the inclusion of individuals from TB specialized hospitals in China. Tuberculosis prevalence surveys, carried out between 2000 and 2010, have reported a significant 31% increase in the proportion of symptom-negative TB patients during that period (17). Similar to a recent meta-analysis by Frascella and colleagues, around half of the prevalent infectious TB burden were subclinical worldwide (18). In a recent nationwide survey conducted in Zambia, 12.5% of untreated confirmed TB patients did not present with clinical symptoms suggestive of active TB, which was significantly lower than our findings (19). This difference is majorly attributed to the diversity of participants across studies. In the latter case, only patients with culture positive or Xpert positive, rather than all active TB cases, were included as subjects in the analysis. In addition, by reviewing the data on repeated surveys, the proportion of subclinical TB slightly increased as TB prevalence declined (19). This trend may be another potential explanation for the low proportion of subclinical TB in Zambia where TB is highly endemic (20). This suggests that passive case finding practice in which TB diagnosis is mainly guided by the presence of cough persisting ≥2 weeks could miss a substantial proportion of the cases, emphasizing the urgent need for the implementation of a more inclusive screening rule at the community level (21).

Individuals with subclinical TB typically carry a considerably lower bacterial load compared with symptomatic individuals, further complicating the diagnosis of TB. We found that the sensitivities of microbiologic tests in subclinical TB were remarkably lower than those in symptomatic TB. A previous study reported that in subclinical TB cases, the nucleic acid amplification test had a positivity rate of 46.2%, while smear microscopy detected a rate of 13.6% (6, 22). This finding was also consistent with our results (40.6% for Xpert and 11.6% for smear microscopy). For subclinical TB, liquid culture exhibited comparable moderate sensitivity to Xpert (40.6% for culture). The poor sensitivities of microbiologic diagnostic tools are mainly attributed to the low mycobacterial load in sputum (23). The modification of current diagnostic procedures may enhance the detection of this paucibacillary population. On the one hand, it always requires a longer time for recovery of tubercle bacilli for sputum samples with low bacterial load. Our previous study has demonstrated that the extended culture after 42 days of incubation could notably improve the recovery rate of liquid culture for paucibacillary specimens (24). On the other hand, the GeneXpert Ultra MTB/RIF assay boasts increased sensitivity relative to Xpert, providing a potential diagnostic method to identify subclinical TB (25).

Conversely, no statistically significant difference was noted in the positivity rate of IGRA between asymptomatic and symptomatic TB patients. Similar to our observations, Theron and co-researchers revealed that in humans with tuberculosis, the strength of the antigen-specific Th1-type immune response was not associated with liquid culture time-to-positive results, smear grade, and Xpert cycle threshold values (26). By contrast, some studies provided some evidence that antigen-specific interferon-γ production by CD4+ T cells correlated with the decrease in bacterial load but did not directly reflect the protection efficacy (27, 28). These contradictory results may be explained by the fact that the high individual variability of Th1-type immune cytokine profiles can lead to different immune responses even with the same bacterial load. Altogether, the lack of correlation between bacterial load and immune response strength provides novel insights showing a potential role of host immune-based diagnostics in clinical management of subclinical TB. More recently, our group has identified antigen-specific CCL8 as a novel biomarker for differentiating latent and active TB (29). Further clinical trials are warranted to investigate its diagnostic accuracy in individuals with subclinical TB.

There is a key question that remains to be answered with respect to which individuals at high risk for subclinical TB should be prioritized for monitoring and treatment (10). In our cohort, we found that the young persons were at high risk for subclinical TB relative to elderly persons (aged >65 years). This trend decreases with aging, whereas the reverse is true for symptomatic TB disease. Our findings are consistent with research showing that individuals below the age of 65 years face an increased likelihood of subclinical TB (8). Within the context of an individual patient, the immunological response may be a determinant for clinical trajectories. In a recent murine experiment, Beer and co-researchers found an impaired interferon response in the aged host, further triggering delayed and insufficient immune response (30). Hence, the elderly individuals with weakened immunity are associated with failure in controlling the actively replicating bacilli, which can in turn accelerate the progression of infection from LTBI to symptomatic TB disease.

In addition, we found an association between employed persons and subclinical TB incidence. Conversely, the unemployed persons were more likely to develop symptomatic TB disease. Compared with unemployed persons, employed individuals are more likely to undergo periodic health examinations. The health examination has been considered an effective measure of identifying disease in asymptomatic people, which may be a plausible explanation for this association. In addition, increasing evidence has implied a role for employment in psychological indicators. Notably, the unemployed persons carry a markedly higher burden of mental illness, and this trend increases with the duration of unemployment (31). Previous data suggest that the presence of stress can provoke changes in a variety of different immune activities, thereby resulting in health impacts (32). Individuals who are more stressed have demonstrated a delay in the immune response to the vaccine (32, 33). Similar results were noted by Freeman and co-researchers who found that psychological stress impairs the function of CD8+ T cell, facilitating the reactivation of latent herpes simplex virus infections (34). Taken together, the compromised immunity of unemployed individuals may be a plausible explanation for greater risk for more severe illness.

To conclude, our results show that approximately 45% of TB patients are subclinical when including individuals from TB-specialized hospitals in China. Microbiologic tests have remarkably lower diagnostic sensitivities for subclinical TB than for symptomatic TB, whereas no statistically significant difference is noted in the positivity rate of IGRA between asymptomatic and symptomatic TB patients, highlighting the potential of antigen-specific immune response in the diagnosis of these asymptomatic individuals. Further clinical trials are warranted to investigate the diagnostic flow chart in individuals with subclinical TB.

## SUPPLEMENTAL MATERIAL

Fig. S1 spectrum.01888-24-s0001.pdf Univariate regression analysis for factors associated with subclinical TB compared with active TB.

ASM does not own the copyrights to Supplemental Material that may be linked to, or accessed through, an article. The authors have granted ASM a non-exclusive, world-wide license to publish the Supplemental Material files. Please contact the corresponding author directly for reuse.
