# Supplementary material for: Diagnostic accuracy of smear microscopy, mycobacterial culture, and GeneXpert MTB/RIF assay for diagnosis of subclinical tuberculosis: a retrospective multicenter study
Source: Microbiol Spectr. 2025 Mar 31;13(5):e01888-24. doi: 10.1128/spectrum.01888-24 (PMC12054030; doi:10.1128/spectrum.01888-24)
Supplement: Fig. S1 — Univariate regression analysis for factors associated with subclinical TB compared with active TB. [file spectrum.01888-24-s0001.pdf]

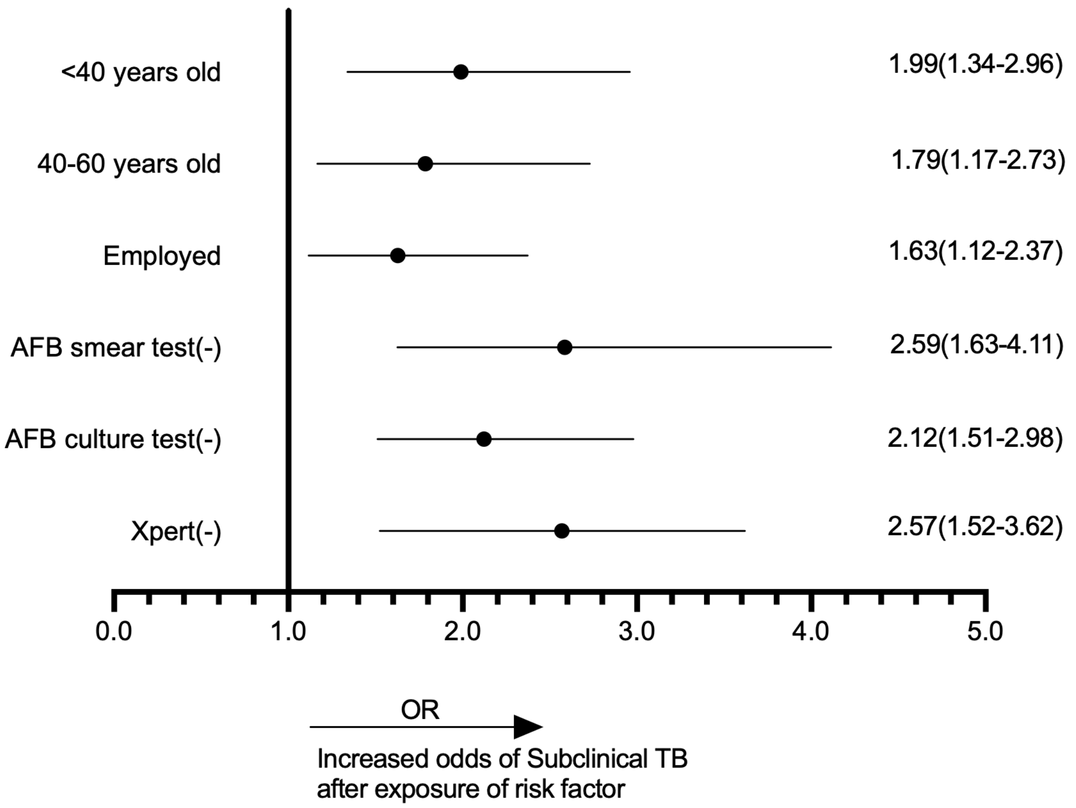

Supplement Figure 1. Univariate regression analysis for factors associated with subclinical TB compared to active TB. The dots denote the summary effect sizes from random effects models and the lines denote 95% CIs for all studies. OR=odds ratio.
